# Increased connectivity of pain matrix in chronic migraine: a resting-state functional MRI study

**DOI:** 10.1186/s10194-019-0986-z

**Published:** 2019-03-25

**Authors:** Mi Ji Lee, Bo-yong Park, Soohyun Cho, Sung Tae Kim, Hyunjin Park, Chin-Sang Chung

**Affiliations:** 1 Department of Neurology, Samsung Medical Center, Sungkyunkwan University School of Medicine, 81 Irwon-Ro, Gangnam-Gu, Seoul, 06351 South Korea; 2 Neuroscience Center, Samsung Medical Center, Seoul, South Korea; 3 Department of Electrical and Computer Engineering, Sungkyunkwan University, Suwon, South Korea; 4 Center for Neuroscience Imaging Research, Institute for Basic Science, Suwon, South Korea; 5 Department of Radiology, Samsung Medical Center, Sungkyunkwan University School of Medicine, Seoul, South Korea; 6 School of Electronic and Electrical Engineering, Sungkyunkwan University, Suwon, South Korea

**Keywords:** Migraine, Chronic migraine, Functional MRI, Neuroimaging, Pain matrix

## Abstract

**Objective:**

To investigate the whole-brain resting-state functional connectivity in patients with chronic migraine (CM) using a data-driven method.

**Methods:**

We prospectively recruited patients with either episodic migraine (EM) or CM aged 18–60 years who visited the headache clinic of the Samsung Medical Center from July 2016 to December 2017. All patients underwent 3 T MRI using an identical scanner. Patients were considered interictal if they did not have a migraine headache at the day and ± 1 days of functional MRI acquisition. Using the group-independent component analysis (ICA), connectivity analysis with a weighted and undirected network model was performed. The between-group differences in degree centrality (DC) values were assessed using 5000 permutation tests corrected with false discovery rate (FDR).

**Results:**

A total of 62 patients (44 EM and 18 CM) were enrolled in this study. Among the seven functionally interpretable spatially independent components (ICs) identified, only one IC, interpreted as the pain matrix, showed a significant between-group difference in DC (CM > EM, *p* = 0.046). This association remained significant after adjustment for age, sex, migraine with aura (MWA), allodynia, depression, and anxiety (*p* = 0.038). The pain matrix was functionally correlated with the hypothalamus (*p* = 0.040, EM > CM) and dorsal raphe nucleus (*p* = 0.039, CM > EM) with different levels of strength in EM and CM.

**Conclusion:**

CM patients have a stronger connectivity in the pain matrix than do EM patients. Functional alteration of the pain network might play a role in migraine chronification.

## Introduction

Migraine is a neurological disorder characterized by episodic headaches associated with nausea, vomiting, and increased sensitivity to external stimuli. Chronic migraine (CM) is a devastating subtype of migraine, which is defined as headache days of 15 or more per month and migraine days of eight or more per month for > 3 months [1]. CM has an estimated prevalence of 1.5% worldwide [2]. CM is more disabling and results in a much higher disease burden than episodic migraine (EM) [3].

About 3% of EMs progress to CMs annually [4]. However, the pathophysiology of migraine chronification is still unknown. Although epidemiological studies revealed risk factors that promote the conversion of EM to CM, biological mechanism of migraine chronification has not been fully elucidated yet, particularly in the absence of medication overuse. Patients can develop CM with or without triggers such as stressful life events, weight gain, and caffeine overuse, which are also common in the lives of healthy people [4, 5]. Therefore, it is likely that a predisposition to migraine chronification exists. To elucidate this, researchers have investigated functional features of the CM brain [6, 7].

To date, studies on functional neuroimaging features of CM are relatively scarce and focused only on predefined brain areas. Schwedt et al. revealed that affective pain regions (anterior insula, amygdala, and PAG) are functionally connected to other brain regions differently in CM patients and normal controls [8]. In studies involving experimental fMRI, CM patients have an enhanced activation of brain regions such as the anterior hypothalamus in response to nociceptive stimuli and nociceptive trigeminal nucleus in response to visual stimuli, compared to normal controls [6, 7]. These studies suggest that distinct functional characteristics of CM exist. However, no study has investigated the whole-brain functional features of CM in comparison to those of EM.

In this study, we aimed to investigate the whole brain resting-state functional connectivity in patients with CM compared to those with EM using a data-driven method. A thorough clinical evaluation and a strict correction for multiple comparisons were performed.

## Methods

### Study subjects

We prospectively recruited new migraine patients who visited the headache clinic of the Samsung Medical Center from July 2016 to December 2017. Patients who were 1) aged 18–60 years, 2) diagnosed with EM or CM, and 3) currently not on migraine preventive medications were included in the study. Patients were excluded if they had 1) medication-overuse headache, 2) chronic pain disorders other than migraine, 3) an alleged diagnosis of major psychiatric disorders such as bipolar affective disorder and schizophrenia, or 4) were currently undergoing treatment for depression or anxiety. Migraine diagnosis was made by two headache specialists (M.J.L. and C.S.C.) according to the International Classification of Headache Disorders, 3rd edition beta version [9].

### Standard protocol approvals, registrations, and patient consents

The Samsung Medical Center Institutional Review Board approved this study. All patients and controls provided written informed consent prior to participation.

### Clinical evaluation

All patients completed a structured questionnaire designed to characterize their headaches. Subsequently, an investigator (M.J.L.) interviewed all patients to verify their responses on the questionnaires. The presence of allodynia was confirmed during the interview. Patients also completed the Allodynia Severity Checklist-12, Patient Health Questionnaire-9 (PHQ-9), and Hospital Anxiety and Depression Scale (HADS) [10–12].

### Functional magnetic resonance image acquisition

All the study subjects underwent magnetic resonance (MR) imaging using a 3 T MR scanner (Achieva; Philips Medical Systems, Best, the Netherlands). T1-weighted structural images with the following imaging parameters were obtained: repetition time (TR) = 9.87 ms; echo time (TE) = 4.59 ms; field of view (FOV) = 240 × 240 mm^2^; number of slices = 360; slice thickness = 0.5 mm; and pixel resolution = 0.5 mm^2^. The imaging parameters for resting-state functional magnetic resonance image (rs-fMRI) are as follows: TR = 3000 ms; TE = 35 ms; flip angle = 90^o^; FOV = 220 × 220 mm^2^; number of slices = 35; slice thickness = 4 mm; pixel resolution = 1.7 mm^2^; and number of volumes = 100.

For all included patients, we assessed the presence and characteristics of headache and the use of acute medications at the day and ± 1 days of fMRI acquisition. Patients were considered interictal if they did not have migraine headache, defined as any headache of moderate to severe intensity, headaches with nausea, vomiting, photophobia, or phonophobia, or headaches that led to the taking of acute migraine medications, at the day and ± 1 days of fMRI acquisition.

### Data preprocessing

Imaging data were preprocessed using fusion of neuroimaging preprocessing (FuNP) pipeline that integrated the AFNI and FSL software [13]. T1-weighted structural MR images were processed by correcting the magnetic field inhomogeneity and removing non-brain tissues. The rs-fMRI data were also processed. The volumes of data obtained during the first 12 s (i.e., four volumes) were discarded to allow the magnetic field to be saturated. The frame-wise displacement (FD) between time series volumes was calculated and the volumes with FD exceeding 0.5 mm were removed [14]. Head motion correction was performed on the remaining time series volumes. Slice timing correction was performed and intensity normalization with a mean value of 10,000 was applied to all the volumes. Nuisance variables such as contributions from white matter, cerebrospinal fluid, head motion, heart, breathing, and the large vein were removed using the FIX software [15]. The low-resolution fMRI data were registered onto the high-resolution T1-weighted data and subsequently onto the Montreal Neurological Institute (MNI) standard space. A band-pass filter with frequency between 0.009 and 0.08 Hz and spatial smoothing with a full width at half maximum of 6 mm was applied.

### Group ICA

The preprocessed rs-fMRI data of all subjects were temporally concatenated, and group independent component analysis (ICA) was performed to automatically generate spatially independent components (ICs) using the FSL MELODIC software [16]. The generated ICs were classified into signal and noise components with two criteria. First, the cross-correlation between the generated ICs and known resting state networks was calculated, and ICs with correlation values less than 0.25 were considered as noise components [17]. Second, signal and noise components were classified by visual inspection based on their spatial map, time series, and frequency spectrum [18, 19]. To identify the functional characteristics of each IC, we performed ‘cognitive decoding’ using Neurosynth software (http://neurosynth.org/) [20]. Neurosynth is an open-source software platform for meta-analyses that enables us to identify relevant specific terms relevant to given activation maps by searching large-scale studies (3228 terms in 14,371 studies, as of March 2019). We applied the cognitive decoding process to the z-statistic map of each IC to identify relevant terms. The cognitive decoding process resulted in correlation values between the z-statistic map of each IC and the activation map of specific terms.

### Main analysis: whole-brain functional connectivity analysis

Connectivity analysis with a weighted and undirected network model was performed. Graph nodes were defined using functionally interpretable ICs (i.e. node = IC) and graph edges were defined as the partial correlation with L2-norm between the time series of different nodes [21]. The correlation values were soft-thresholded to avoid binarizing edge weights [22, 23]. The soft-thresholded correlation values were transformed to *z*-values using Fisher’s *r*-to-*z* transformation. Degree centrality (DC), which measures the importance of a given node, was calculated for each node by summing all edge weights connected to a given node [24]. DC values of each node were used for identifying differences between the EM and CM groups.

### Secondary analysis: connectivity with the hypothalamus, dorsal raphe nucleus, and periaqueductal gray

**Fig. 1:**
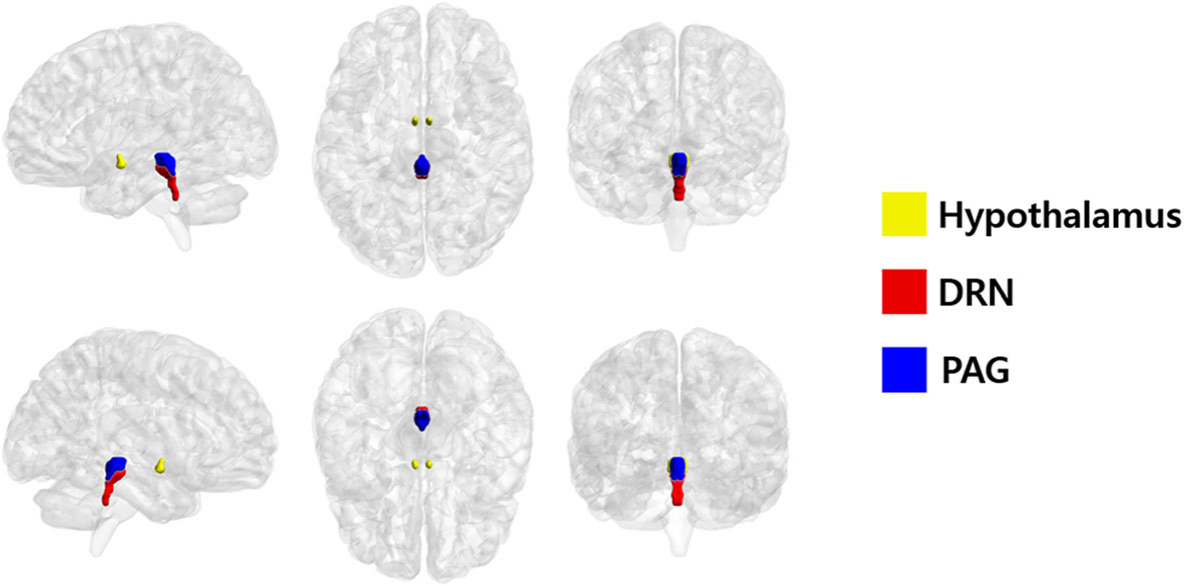
Region of interest segmentation results. The hypothalamus, dorsal raphe nuclei (DRN), and periaqueductal gray (PAG) were segmented on a three-dimensional brain atlas

After identifying brain networks which showed significant between-group differences, we tested whether the identified network was relevant to migraine pathophysiology. We defined three regions of interest (ROIs): the hypothalamus, dorsal raphe nucleus (DRN), and periaqueductal gray (PAG). The hypothalamus and PAG were manually drawn, while the DRN was defined using the Harvard ascending arousal network atlas via image co-registration (Fig. 1). [25] The centroid coordinates of ROIs in the MNI standard space were consistent with previous studies (hypothalamus: x = 0, y = − 4, z = − 9; PAG: x = 1, y = − 31, z = − 9; DRN: x = 1, y = − 32, z = − 17) [26–29]. The time series were extracted from each ROI and their correlation with the time series of the identified functional network in the main analysis were computed between all possible pairs. The correlation values were transformed to *z*-values using Fisher’s *r*-to-*z* transformation. The *z*-transformed correlation values were used for identifying differences between the EM and CM groups.

### Statistical analysis

Clinical variables were compared between EM and CM groups using the Chi-square test, Fisher’s exact test, or Mann-Whitney tests. The differences in DC values between EM and CM groups were assessed using permutation tests followed by false discovery rate (FDR) correction [30, 31]. Subjects were randomly assigned to the EM and CM groups 5000 times, and a null distribution was constructed. The ICs with DC values outside 95% of the null distribution were considered significant ICs with significant between-group differences. The *p*-values were further corrected using FDR (*p* < 0.05, corrected) [30]. Multivariable linear regression analysis with adjustment for age, sex, presence of aura, allodynia, depression (PHQ-9 scores ≥8), anxiety (HADS-A scores of ≥8), disease duration, headache intensity, and acute antimigraine drug use/month was performed. Pearson’s correlation analysis between the strength of identified brain networks and monthly headache days was performed to assess if the group difference was the consequence of frequent headaches. The correlation analysis was also performed between the strength of identified brain networks and clinical variables such as patients’ disease duration, allodynia, anxiety, and depression scores. Interaction analysis was performed to determine a possible modifying effect of the presence of depression and anxiety. Statistical analysis was performed using MATLAB 2017a (Mathworks Inc., Natick, MA, USA) and SPSS software (IBM-SPSS. Chicago, IL, USA).

## Results

### Study subjects

Among 86 eligible patients, 64 (45 EM and 19 CM) underwent the interictal study. Among them, two (1 EM and 1 CM) were excluded from the analysis because of poor image quality. Finally, data from 62 patients (44 EM and 18 CM) were used for the analysis. Demographics and characteristics of patients are summarized in Table 1.

**Table 1:** Demographics and characteristics of study participants

	Episodic migraine (*n* = 44)	Chronic migraine (*n* = 18)	P
Age (range)	40 ± 10.2 (22–57)	41.4 ± 10.9 (19–55)	0.622
Female sex	36 (81.82%)	11 (61.11%)	0.084
Disease duration, y	12.0 ± 9.0	12.9 ± 9.9	0.822
Headache days per month	6.3 ± 3.6	23 ± 5.9	< 0.001
Moderate/severe headache days per month	4.3 ± 2.7	13.3 ± 7.9	< 0.001
Migraine with aura	4 (9.09%)	4 (22.22%)	0.214
Allodynia	8 (18.18%)	5 (27.78%)	0.400
Anxiety	15 (34.09%)	9 (50%)	0.243
Depression	6 (13.64%)	6 (33.33%)	0.075

Data are presented as mean ± SD or N (%) unless otherwise specified

### Functional network identification

**Fig. 2:**
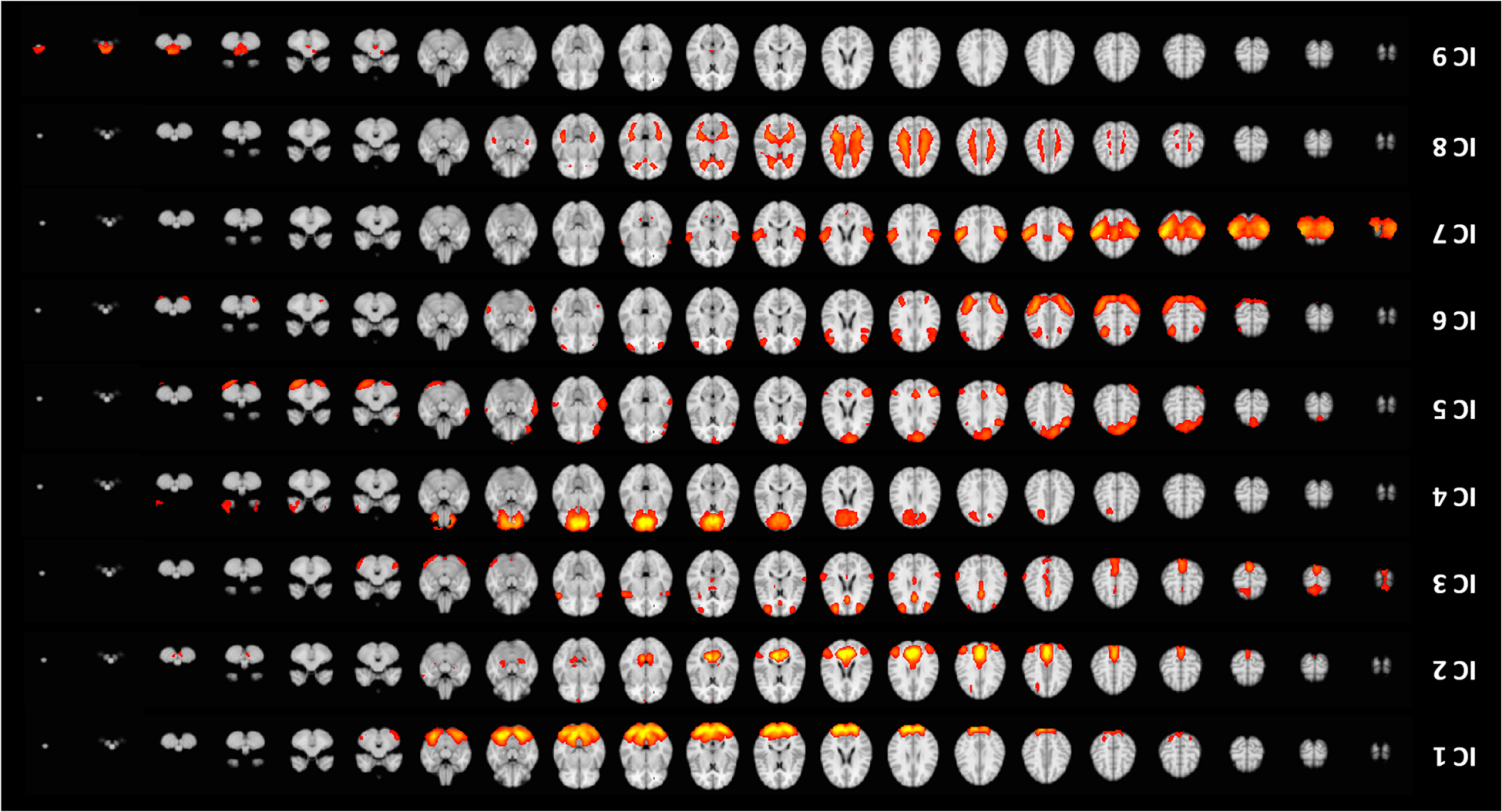
Resting-state networks identified using independent component analysis. Nine automatically generated independent components (ICs). ICs 8 and 9 were considered as noise components and were therefore excluded. ICs 1 to 7 are functionally interpretable ICs

**Fig. 3:**
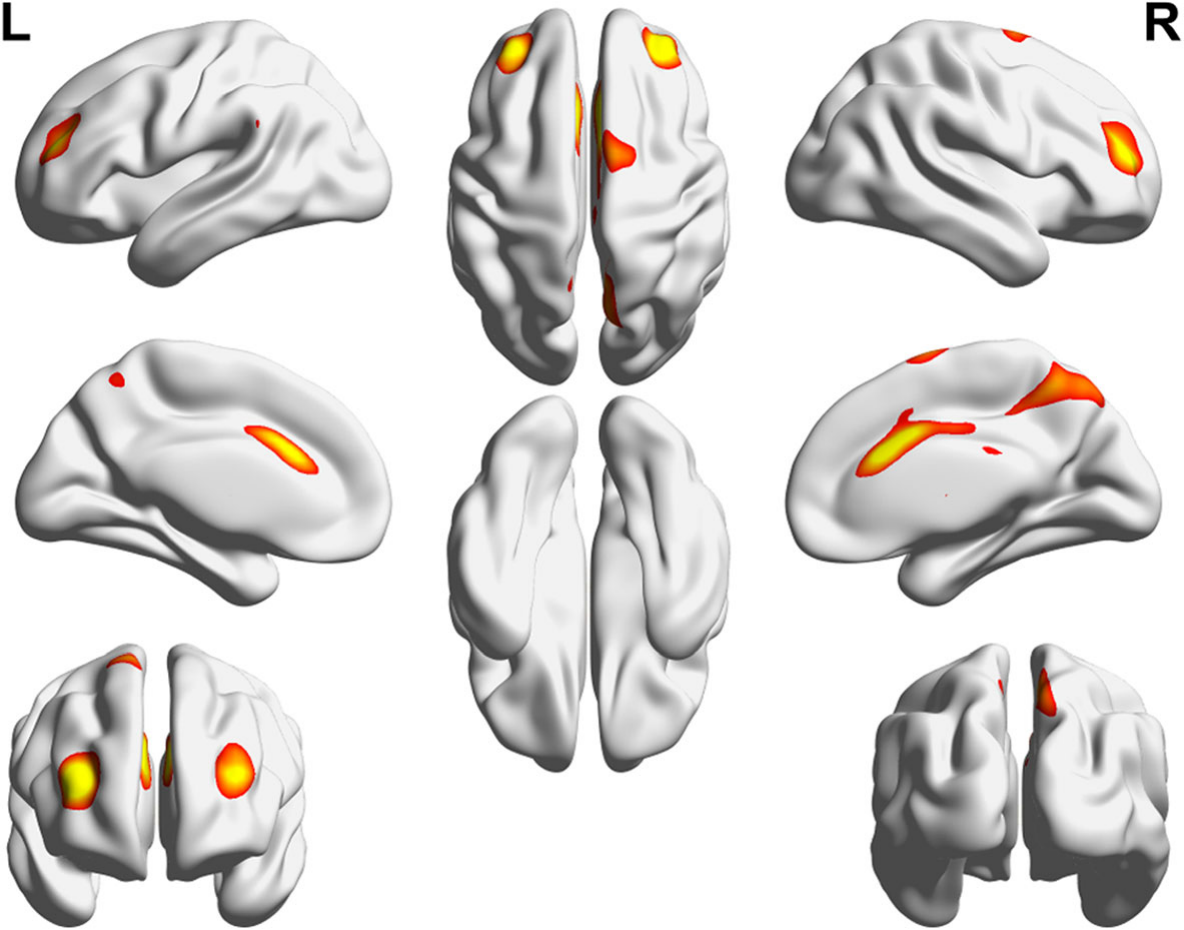
Pain matrix. The functional network (IC 3) included the dorsolateral prefrontal cortex, anterior insular cortex, anterior cingulate cortex, thalamus, and precuneus, suggesting a pain matrix

The group-ICA approach automatically generated nine ICs (Fig. 2). Two ICs (white matter and noise components) were excluded from further analyses. Finally, seven functionally interpretable ICs were identified. All but IC 3 were compatible with known resting-state functional networks: IC 1 (visual network) comprises the lingual gyrus, and superior- and inferior- occipital cortices; IC 2 (default mode network) comprises the posterior cingulate cortex and precuneus; IC 4 (executive control network) comprises the medial prefrontal cortex, orbitofrontal cortex, and anterior cingulate cortex (ACC); IC 5 (frontoparietal network) comprises the superior frontal cortex, angular gyrus, and posterior cingulate cortex; IC 6 (frontoparietal network) comprises the orbitofrontal cortex, and superior and inferior parietal gyri; and IC 7 (sensorimotor network) comprises the pre-central and post-central gyri and paracentral lobule. Major components of IC 3 included the dorsolateral prefrontal cortex (DLPFC), anterior insula, ACC, thalamus, and precuneus (Fig. 3). IC 3 also included supramarginal gyrus, planum temporale, premotor cortex, and cerebellum. Based on the cognitive decoding process using Neurosynth, we identified IC 3 as the pain matrix (Table 2).

**Table 2:** The results of the cognitive decoding process of the ICs using Neurosynth software

ICs	Terms^a^	Correlation value
1	Visual	0.610
	Sighted	0.328
	Lingual	0.311
2	Default	0.404
	Autobiographical	0.310
	Episodic	0.302
3	Response inhibition	0.185
	Pain	0.170
	Painful	0.161
4	Value	0.197
	Reward	0.178
	Default	0.177
5	Mind	0.337
	Theory mind	0.328
	Default	0.323
6	Working memory	0.459
	Calculation	0.393
	Tasks	0.388
7	Somatosensory	0.624
	Sensorimotor	0.613
	Primary motor	0.6

^a^The terms with the top three correlation values were reported

### Differences in functional connectivity

**Fig. 4:**
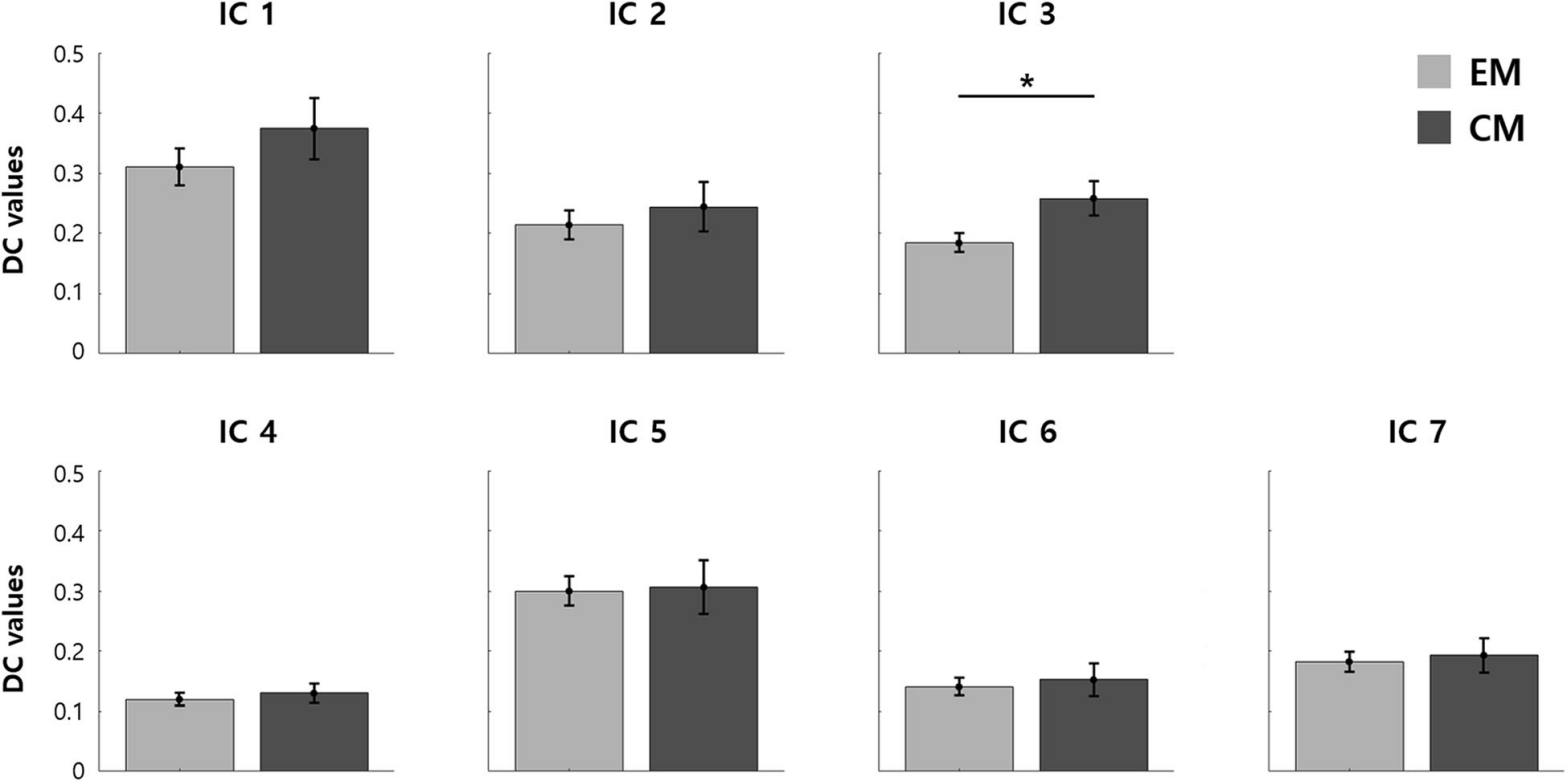
Group comparison among identified resting-state networks. DC values were compared between EM and CM by using permutation tests with FDR. Each bar and error bar represent the mean and standard errors of mean, respectively

Figure 4 shows between-group differences in DC values among identified ICs. A significant between-group difference was found only in IC 3 (pain matrix). Patients with CM showed stronger connectivity in terms of DC in the pain matrix than those with EM (uncorrected *p* = 0.0066 and FDR-corrected *p* = 0.0462). This between-group difference remained significant after adjustment for covariates such as age, sex, migraine with aura (MWA), allodynia, depression, anxiety, disease duration, headache intensity, and acute antimigraine drug use/month (Table 3).

**Table 3:** Univariable and multivariable analysis of between-group difference in the connectivity of the pain matrix

	*P* value^*^
Univariable	0.0462
Multivariable	
Adjusted for age, sex, MWA	0.0420
Adjusted for age, sex, MWA, allodynia	0.0238
Adjusted for age, sex, MWA, depression, anxiety	0.0210
Adjusted for age, sex, MWA, allodynia, depression, anxiety	0.0378
Adjusted for age, sex, MWA, allodynia, depression, anxiety, disease duration	0.0283
Adjusted for age, sex, MWA, allodynia, depression, anxiety, disease duration, headache intensity	0.0300
Adjusted for age, sex, MWA, allodynia, depression, anxiety, disease duration, headache intensity, acute antimigraine drug use/month	0.0291

*MWA* = migraine with aura

^*^P values were corrected for multiple comparisons by using the false discovery rate (FDR) correction

To investigate the relative importance of the sub-regions of the pain matrix, we calculated mean z-statistic values of the ICA weights from the sub-regions within the pain matrix. The sub-region with the highest z-statistic value was ACC (= 3.693) followed by precuneus (= 3.650), DLPFC (= 3.548), premotor cortex (= 3.004), supramarginal gyrus (= 2.956), planum temporale (= 2.937), cerebellum (= 2.825), anterior insula (= 2.789), and thalamus (= 2.419). The results showed that ACC was the most important sub-region in the IC 3 and might be the largest contributing factor to explain the between-group differences between EM and CM groups.

### Clinical correlates of the pain matrix connectivity

We performed correlation analyses between clinical variables and the connectivity (i.e. DC values) of pain matrix. No significant correlation was found between the pain matrix connectivity and headache days (r = 0.0444, *p* = 0.7321), HADS-D score (r = 0.1080, *p* = 0.4239), HADS-A score (r = 0.0322, *p* = 0.8119), PHQ-9 score (r = − 0.0400, *p* = 0.7638), ASC-12 score (r = 0.0306, *p* = 0.8136), and disease duration (r = − 0.0910, *p* = 0.4818).

Neither depression nor anxiety modified the association between CM and the pain matrix connectivity (P for interaction = 0.479 and 0.425, respectively). The presence of mild non-migrainous headache on the day of fMRI acquisition also did not modify this association (P for interaction = 0.372).

### Functional correlates of the pain matrix connectivity

**Fig. 5:**
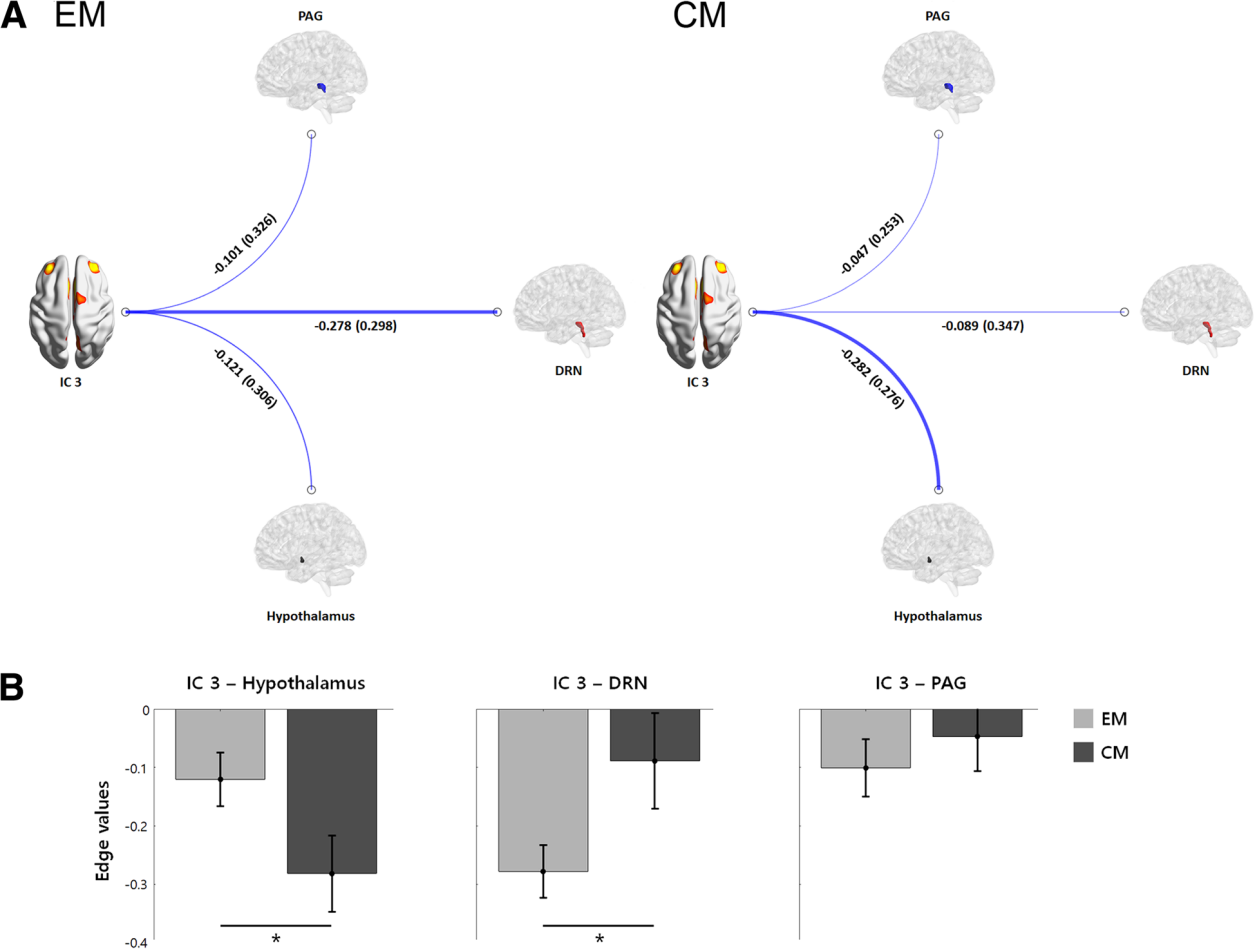
Functional connectivity of IC 3 (pain matrix) with the hypothalamus, DRN, and PAG. **a** Functional connectivity of IC 3 with the hypothalamus, dorsal raphe nuclei (DRN), and periaqueductal gray (PAG) are illustrated. The mean (SD) of edge values are shown and the width of the lines indicate the magnitude of edge weights. **b** Between-group analysis of connectivity between IC 3 and each region are summarized. Patients with CM showed a stronger connectivity between IC 3 and the hypothalamus (FDR-corrected *p* = 0.0399), whereas the connectivity between the DRN and IC 3 was stronger in patients with EM (FDR-corrected *p* = 0.0390). No between-group difference in the connectivity between IC 3 and the PAG were noted (FDR-corrected *p* = 0.2738)

Figure 5 shows connectivity between key regions involved in migraine pathophysiology and the pain matrix. The strength of the functional connection between the pain matrix and the hypothalamus (CM > EM, FDR-corrected *p* = 0.0399) and DRN (EM > CM, FDR-corrected *p* = 0.0390) was different between groups. No significant between-group difference was found in the connectivity between the pain matrix and PAG (FDR-corrected *p* = 0.2738).

## Discussion

In this study, we found that 1) the connectivity in the pain matrix differed between EM and CM patients; 2) the pain matrix connectivity was not correlated with headache frequency or psychiatric comorbidities; and 3) the strength of the functional connection between the pain matrix and the hypothalamus and DRN was different between EM and CM groups. An enhanced connectivity of the pain matrix may play a role in migraine chronification.

### Data-driven vs. ROI-based method

To date, the neural mechanism underlying migraine chronification is still unknown. To unveil functional characteristics of CM, functional neuroimaging is used for research. Using a resting-state or task-specific functional MRI, specific brain regions were tested with a priori hypothesis using ROI-based approaches [6–8]. However, no study has compared whole-brain connectivity features between CM and EM. In this study, the group ICA approach was adopted to define large-scale brain networks. The major advantage of using group ICA over pre-defined atlases is that it is a data-driven approach. There are many existing pre-defined atlases including Brodmann areas, automated anatomical labeling (AAL), and Harvard-Oxford atlases [32, 33]. However, the atlases were constructed using different pools of subjects and each atlas provides a different number of brain regions, which might reduce the reproducibility of the neuroimaging studies. In addition, the pre-defined atlases might not reflect the functional characteristics adequately since they were derived from a different set of subjects. The group-ICA approach yields brain networks that share similar activity patterns among the patients and thus, reflects the functional characteristics of the data more robustly than the atlas-based approach.

### Pain matrix in CM

In this study, a functional network (pain matrix), which comprised the ACC, anterior insulae, thalami, DLPFC, precuneus, supramarginal gyri, and cerebellum, differed between EM and CM in its functional connectivity. The concept of a pain matrix has been challenged by studies which reported that similar areas are activated in response to non-nociceptive stimuli [34]. In addition, the pain matrix overlaps with the salience circuit, which is implicated in chronic pain processing [35]. However, in our study, the pain matrix additionally involves areas such as the DLPFC and supramarginal gyri, which are considered as major components of the central executive network. In addition to the salience circuit, these regions overlap with areas involved in pain experience (the ACC, anterior insular, and thalamus) [36], cognitive modulation of pain sensitivity (precuneus) [37], pain expectation (DLPFC, insula, ACC, globus pallidus, putamen, thalamus, and cerebellum) [38], and pain catastrophizing on mild pain (ACC, insula, DLPFC, precuneus, thalamus, putamen, inferior parietal lobule, parahippocampal gyrus) [39]. In concordance with the results of a previous study using an experimental fMRI paradigm which reported that migraineurs have enhanced pain-induced activity of the pain matrix [40], we observed that the functional connectivity of pain matrix was more greater in patients with CM.

The matrix identified in this study is different from the functional MRI markers of acute pain which include somatosensory areas (S1 and S2) and PAG [41]. CM can be either a predisposition to or state of frequent headaches. However, CM brains did not show markers of continuing acute pain. Instead, the insulae and ACC, which play a major role in chronic pain [42], as well as other pain-related and cognitive areas, had a stronger functional connectivity in CM. Our data suggest that a stronger connectivity of the pain matrix is a characteristic of the CM brain, which might play a major role in migraine chronification.

### Different functional features between CM and EM

Whether EM and CM are different disorders or in a single continuum has been debated for a long time. In this resting-state fMRI study, we suggest that CM has functional characteristics distinct from EM. Previously, only a limited number of studies investigated resting-state fMRI features of CM and most of them focused on specific structures of interest (e.g. amygdala, insula, and ACC) [8, 43]. Our findings are in line with previous study results on involvement of limbic structures in CM, especially ACC and insular cortex. Although our cross-sectional study is not suitable for proving any causal relationship, the functional connectivity did not correlate with headache frequencies or psychologic comorbidities, suggesting that our findings are not a consequence of them but a predisposition to migraine chronification. We are currently conducting a prospective fMRI study to test the change of brain functional characteristics in association with disease courses in patients with migraine (ClinicalTrials.gov Identifier: NCT03487978).

### Comparison with functional neuroimaging of chronic pain

fMRI has been used in research on chronic pain disorders in several previous studies. The prefrontal cortices, insulae, and ACCs are reported to be activated in most chronic pain disorders. However, conflicting results on the resting-state connectivity of networks comprising the aforementioned areas exist. Specifically, a greater connectivity was reported between the default mode network and ACC in patients with diabetic neuropathic pain [44]; between the default mode network and insular cortices in patients with fibromyalgia [45]; in the salience network, central executive network, and default mode network in pediatric patients with complex regional pain syndrome (CRPS) [46]; and between the right insular cortices and cingulate gyri in patients with fibromyalgia [47]; while reduced connectivity among the medial prefrontal cortex, insular cortex, and ACC were found in patients with chronic pain disorders such as CRPS, knee osteoarthritis, and chronic back pain [48].

Clinically, CM differs from other chronic pain disorders because the “chronicity” in migraine does not imply persistent pain but increased days of headache, which is a combination of frequent episodic attacks (a function of the headache generator) and lower tendency toward clear remission (a function of either an enhanced pain signaling or decreased pain modulation). Our main analysis showed that an increased connectivity within the pain matrix may play a role in migraine chronification. However, the role of the migraine generator should not be overlooked because small structures such as the hypothalamus and PAG might have been missed by the whole-brain, data-driven approach used in our study. Thus, we further tested three structures involved in migraine pathophysiology: the hypothalamus, a migraine generator; PAG, a pain modulatory center; and DRN, the main serotonergic center activated during migraine attack [49–51]. As a result, patients with CM showed an increased connectivity between the pain matrix and hypothalamus compared to those with EM, while the connectivity between the pain matrix and DRN was weaker in CM patients. Taken together with a recent report of increased hypothalamic activation to painful stimuli in CM [6], we suggest that the hypothalamus is more easily activated by external stimuli and strongly connected to the pain matrix in patients with CM, while the brain connectivity between the pain matrix and serotonergic system in patients with CM is relatively weak. Our data suggest that the pain matrix is another key player in CM pathophysiology.

Our study has several strengths. First, we carefully defined CM and performed interictal fMRI imaging in patients with CM. Second, a data-driven method was used, and a strict statistical correction was performed to avoid pitfalls during multiple comparisons. The limitations of our study include 1) the small number of participants, 2) the lack of normal controls, and 3) the cross-sectional nature of the study. To overcome these limitations, we are currently conducting a longitudinal fMRI study in migraineurs and controls to study the effects of the disease and time on fMRI changes. In addition, we could not investigate some important structures (e.g. dorsal pons) and nuclei involved in migraine pathophysiology (e.g., the nucleus raphe magnus and superior salivatory nucleus) because of non-availability of reliable atlases.

## Conclusions

In conclusion, CM has an enhanced functional connectivity of the pain matrix which has a different functional connection to hypothalamus and DRN compared to EM patients. Functional alteration of the pain network might play a role in migraine chronification.

## Abbreviations

AAL: Automated anatomical labeling
CM: Chronic migraine
CRPS: Complex regional pain syndrome
DC: Degree centrality
DLPFC: Dorsolateral prefrontal cortex
DRN: Dorsal raphe nucleus
EM: Episodic migraine
FD: Frame-wise displacement
FDR: False discovery rate
FOV: Field of view
HADS: Hospital Anxiety and Depression Scale
IC: Independent component
ICA: Independent component analysis
MNI: Montreal Neurological Institute
MR: Magnetic resonance
MWA: Migraine with aura
PAG: Periaqueductal gray
PHQ-9: Patient Health Questionnaire-9
ROI: Region of interest
rs-fMRI: Resting-state functional magnetic resonance image
TE: Echo time
TR: Repetition time
